# Signaling network of dendritic cells in response to pathogens: a community-input supported knowledgebase

**DOI:** 10.1186/1752-0509-4-137

**Published:** 2010-10-07

**Authors:** Sonali Patil, Hanna Pincas, Jeremy Seto, German Nudelman, Irina Nudelman, Stuart C Sealfon

**Affiliations:** 1 Center for Translational Systems Biology and Department of Neurology, Mount Sinai School of Medicine, New York, NY 10029, USA; 2 Computational Biology Program, Courant Institute, New York University, New York, NY 10012, USA

## Abstract

**Background:**

Dendritic cells are antigen-presenting cells that play an essential role in linking the innate and adaptive immune systems. Much research has focused on the signaling pathways triggered upon infection of dendritic cells by various pathogens. The high level of activity in the field makes it desirable to have a pathway-based resource to access the information in the literature. Current pathway diagrams lack either comprehensiveness, or an open-access editorial interface. Hence, there is a need for a dependable, expertly curated knowledgebase that integrates this information into a map of signaling networks.

**Description:**

We have built a detailed diagram of the dendritic cell signaling network, with the goal of providing researchers with a valuable resource and a facile method for community input. Network construction has relied on comprehensive review of the literature and regular updates. The diagram includes detailed depictions of pathways activated downstream of different pathogen recognition receptors such as Toll-like receptors, retinoic acid-inducible gene-I-like receptors, C-type lectin receptors and nucleotide-binding oligomerization domain-like receptors. Initially assembled using CellDesigner software, it provides an annotated graphical representation of interactions stored in Systems Biology Mark-up Language. The network, which comprises 249 nodes and 213 edges, has been web-published through the Biological Pathway Publisher software suite. Nodes are annotated with PubMed references and gene-related information, and linked to a public wiki, providing a discussion forum for updates and corrections. To gain more insight into regulatory patterns of dendritic cell signaling, we analyzed the network using graph-theory methods: bifan, feedforward and multi-input convergence motifs were enriched. This emphasis on activating control mechanisms is consonant with a network that subserves persistent and coordinated responses to pathogen detection.

**Conclusions:**

This map represents a navigable aid for presenting a consensus view of the current knowledge on dendritic cell signaling that can be continuously improved through contributions of research community experts. Because the map is available in a machine readable format, it can be edited and may assist researchers in data analysis. Furthermore, the availability of a comprehensive knowledgebase might help further research in this area such as vaccine development. The dendritic cell signaling knowledgebase is accessible at http://tsb.mssm.edu/pathwayPublisher/DC_pathway/DC_pathway_index.html.

## Background

The innate immune system represents the first line of defense against attack by viral, bacterial, and parasitic infections. Dendritic cells (DCs), which are found in abundance in peripheral tissues such as skin, lung, and mucosal surfaces, act as a bridge between the innate and adaptive immune systems: recognition of a 'danger' signal initiates the maturation of DCs, which ultimately activate cells of the adaptive arm of the immune system, B and T cells [1-3]. DCs express receptors that recognize and bind a large array of epitopes or antigens common to many bacterial or viral pathogens; once an antigen is recognized, it is internalized, processed, and presented at the cell surface in association with molecules of the major histocompatibility complex (MHC). DC maturation is characterized by up-regulation of the MHC molecules, production of cytokines, chemokines and co-stimulatory molecules, and migration of DCs to lymphoid tissues, i.e. the spleen and the lymph nodes (for review, see [3,4]). Research efforts have aimed at understanding the DC signaling and effector pathways that direct this cell's crucial role in immunity. A graphical representation of those signaling pathways as a biological system would provide an easily accessible, integrated view of the literature in this field to the scientific community.

DCs detect pathogens via pattern recognition receptors (PRRs), which recognize various molecular structures referred to as pathogen-associated molecular patterns (PAMPs), e.g. lipopolysaccharides, lipoteichoic acids, flagellin and nucleic acids. Membrane-associated PRRs, like the Toll-like receptors (TLRs) and C-type lectin receptors (CLRs) respond to extracellular pathogens, while cytosolic PRRs, including RIG-I-like receptors (RLRs) and NOD-like receptors (NLRs) sense intracellular pathogens [5-7]. Pathogen recognition activates an intracellular signaling cascade, which results in the expression of type I interferons (IFNs), as well as other inflammatory response genes. Secreted IFNs bind to cell surface receptors and activate the JAK-STAT pathway in an autocrine and paracrine fashion [8,9]. A resource that facilitates access to information on the molecular networks that underlie DC signaling responses to various pathogens would assist research on antibacterial and antiviral therapy. Furthermore, it might benefit the development of DC vaccines against cancers and autoimmune diseases, as manipulating DCs *in vitro *and rendering them responsive to tumor antigens may lead to tumor regression [10]. Traditional representations of molecular pathways may be found in reviews. A web-based pathway diagram complements these reviews by giving more direct access to continually updated literature information in a pathway format. Also a pathway-based resource can be used directly for computational studies. SBML is a computer-readable format for representing models of biological processes [11]. Therefore, an optimal pathway diagram should focus on a whole biological system rather than a part of it, comply with a standard format, such as the Systems Biology Mark-up Language (SBML; http://sbml.org/), and be accessible to the community for updates and corrections.

Current databases of signaling networks of the innate immune response include free online resources such as the Kyoto Encyclopedia of Genes and Genomes (KEGG) Pathway database http://www.genome.jp/kegg/pathway.html[12], Reactome, which is a curated knowledgebase of biological pathways http://www.reactome.org/cgi-bin/frontpage?DB=gk_current[13], Science's Signal Transduction Knowledge Environment (STKE; http://stke.sciencemag.org/cm/) biological pathways database, and Ingenuity Systems, a commercial subscription-based knowledgebase. The KEGG pathways do not integrate all PRRs, but rather depict each type of PRR-derived pathway separately and with little detail. Although Reactome is abundantly annotated and organism-specific, it does not provide cell-type specific information. STKE features 19 immunity-related pathways, the majority of which, however, have not been updated in recent years. Similar to Reactome, Ingenuity Systems offers a great diversity of annotations, including literature references from various biological models and many other database resources, yet it presents a fairly basic version of each signaling network and is not cell-type specific. In contrast, the group of Kitano [14] constructed a comprehensive map of TLRs and interleukin 1 receptor signaling networks based on published literature; the TLR pathway map, created in CellDesigner [15], is comprised of 652 species and 444 reactions and complies with SBML. However, it lacks a means for community-wide feedback, which would considerably help experts in the field to directly participate in the map update, and is not cell-type specific.

Based on a manual curation of the published literature, we have assembled an extensive and detailed map of the signaling pathways involved in DC response to pathogens, as described in human DCs and mouse models. The DC pathway map, which is web-accessible, includes the following annotations: a list of interactions, GeneIDs, PubMed IDs (PMIDs), along with summary notes. In order to provide a discussion forum for the community and an opportunity for direct feedback and contribution, we have linked the DC map to a public wiki. Thus, it should represent a valuable resource for the research community, and conceivably initiate a community-wide interactive process. Additionally, using computational methods we delineated the regulatory motifs that are present in the DC signaling network.

## Construction and content

**Figure 1:**
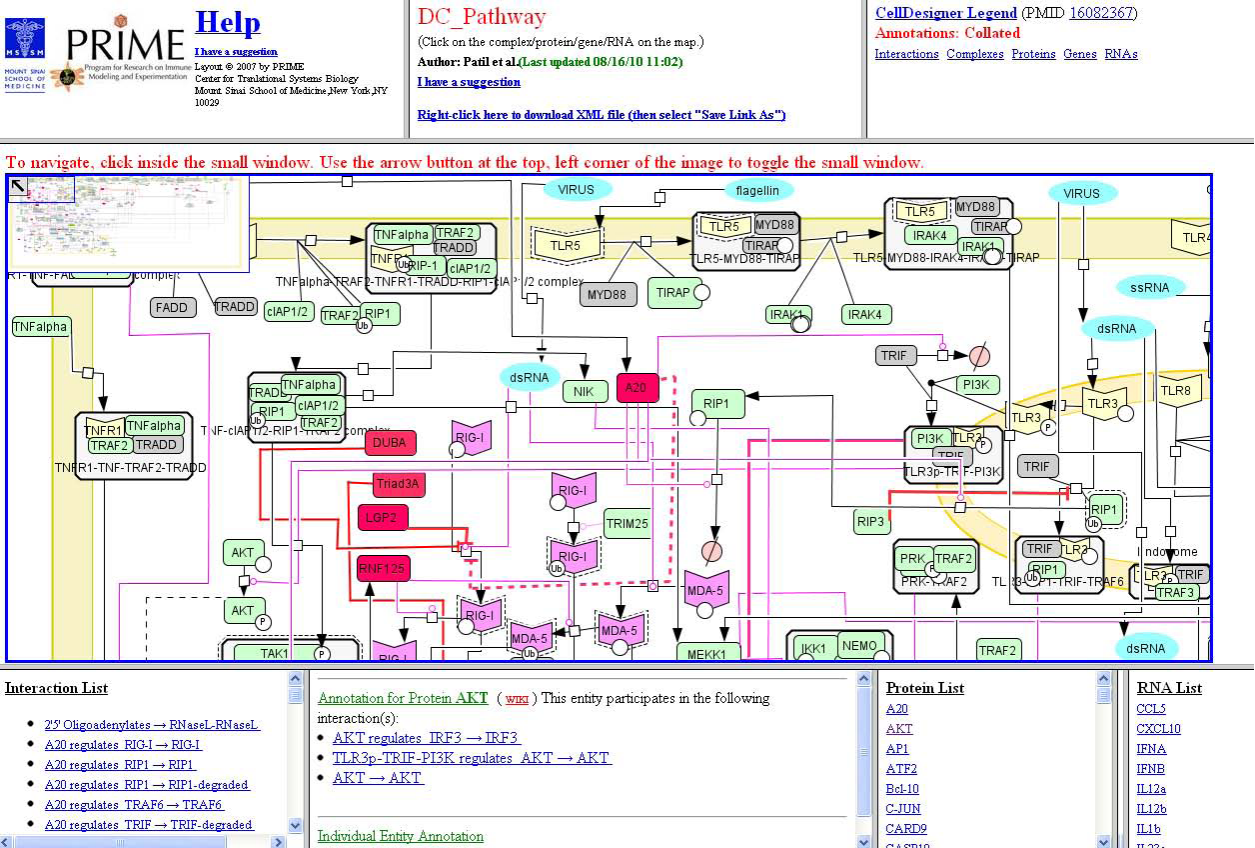
**Web-accessible DC pathway navigator**. Snapshot of the pathway navigator that displays a selected view of the DC signaling map. Entities are clickable, such that corresponding annotations, namely the interactions in which those entities are involved, and the related PMIDs are displayed in the bottom left-hand frame. Additionally, other frames include an Interaction list, a Protein list, a Gene list, and an RNA list. Hyperlinks to the relevant NCBI Entrez Gene page(s) and DC wiki pages are also provided. A zoom rectangle located in the upper left corner of the image facilitates navigation through the pathway.

To summarize the complexity of the pathogen recognition response in DCs, we have developed a map of signaling events occurring in those cells upon viral and bacterial infection. This map was assembled in CellDesigner, a free process diagram editor for gene-regulatory and biochemical networks http://celldesigner.org/; it was then web-published using BioPP, a software suite which converts CellDesigner-SBML formatted pathways into a web-viewable format [16]. Hence, this knowledgebase is deposited into a public repository endowed with a pathway navigator, which facilitates browsing through the nodes and entities of the uploaded pathway. Each entity in the network is annotated with a complete list of interactions in which it participates, and PMIDs supporting those interactions (Figure 1). Entities are also linked to NCBI Entrez Gene pages and to their respective wiki pages. Furthermore, users may download the DC signaling pathway diagram from the BioPP website in an xml format, which allows them to edit and/or expand it in CellDesigner, based upon their own experimental data or knowledge.

At present, the DC signaling network consolidates manually curated information from 167 peer-reviewed journal articles. Target publications are mainly reports on human DCs and mouse models, as well as studies in heterologous expression systems. In particular, as the main interest of our research group is monocyte-derived DCs obtained from human blood donors, namely conventional DCs, we have not yet included plasmacytoid DCs in the knowledgebase. To avoid adding more visual complexity to the diagram, we have chosen not to depict response genes, but rather represent their respective RNAs. For simplicity, transcription factors and other regulatory complexes are directly connected to the RNAs of the response genes they activate. Additionally, the molecular machinery required for the transportation of transcription factors from the cytoplasm to the nucleus and *vice versa *has been omitted. The network consists of 249 entities or nodes and 213 reactions or edges. Among the 118 protein species, 20 were classified as receptors and 2 as truncated proteins, the remainder being comprised of intracellular proteins as well as transcription factors. The reactions can be classified into 122 state transitions (which include catalysis reactions), 9 heterodimer associations, 4 dissociations, 36 transcriptional activations, 8 unknown transitions, 8 transport reactions, 21 inhibitions, and 4 translations.

### Main structural features of the map

**Figure 2:**
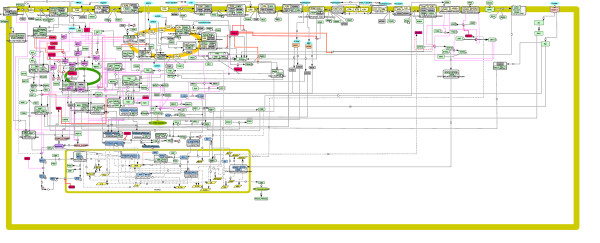
**Snapshot of the DC signaling pathway map**. This map was created using CellDesigner ver.4.0.1, ver.4.0.α, and ver.4.1β http://celldesigner.org/. The main symbols used may slightly differ from those implemented by CellDesigner ver.4.0.1 and ver.4.0.α, as indicated. Interactions are color-coded: black filled arrows, stimulatory reactions; red bar-headed lines, inhibitory reactions; black dashed and double-dotted lines, transcriptional activation reactions (instead of transcription reactions); pink round-headed lines, catalysis reactions; black filled arrows with a bar, transport reactions. The presence of a question mark signifies that, whether the reactions are direct or indirect, is unknown. Translation reactions are represented by dashed and single-dotted lines. Modification states of proteins, i.e. phosphorylation and ubiquitination, are symbolized by P and Ub, respectively. Entities that are bordered with a dotted frame are considered in an active state. The following cellular compartments are illustrated on the diagram, as indicated: cytoplasm, endosome, mitochondria, and nucleus. The color-coding of entities was put together by us: pathogens and PAMPs in turquoise, TLRs in yellow, RLRs in purple, CLRs in pink, NLRs in orange, adaptors in grey, transcription factors in light blue, kinases in light green, and negative regulators in red. The map can be more easily viewed on the web at http://tsb.mssm.edu/pathwayPublisher/DC_pathway/DC_pathway_index.html.

**Figure 3:**
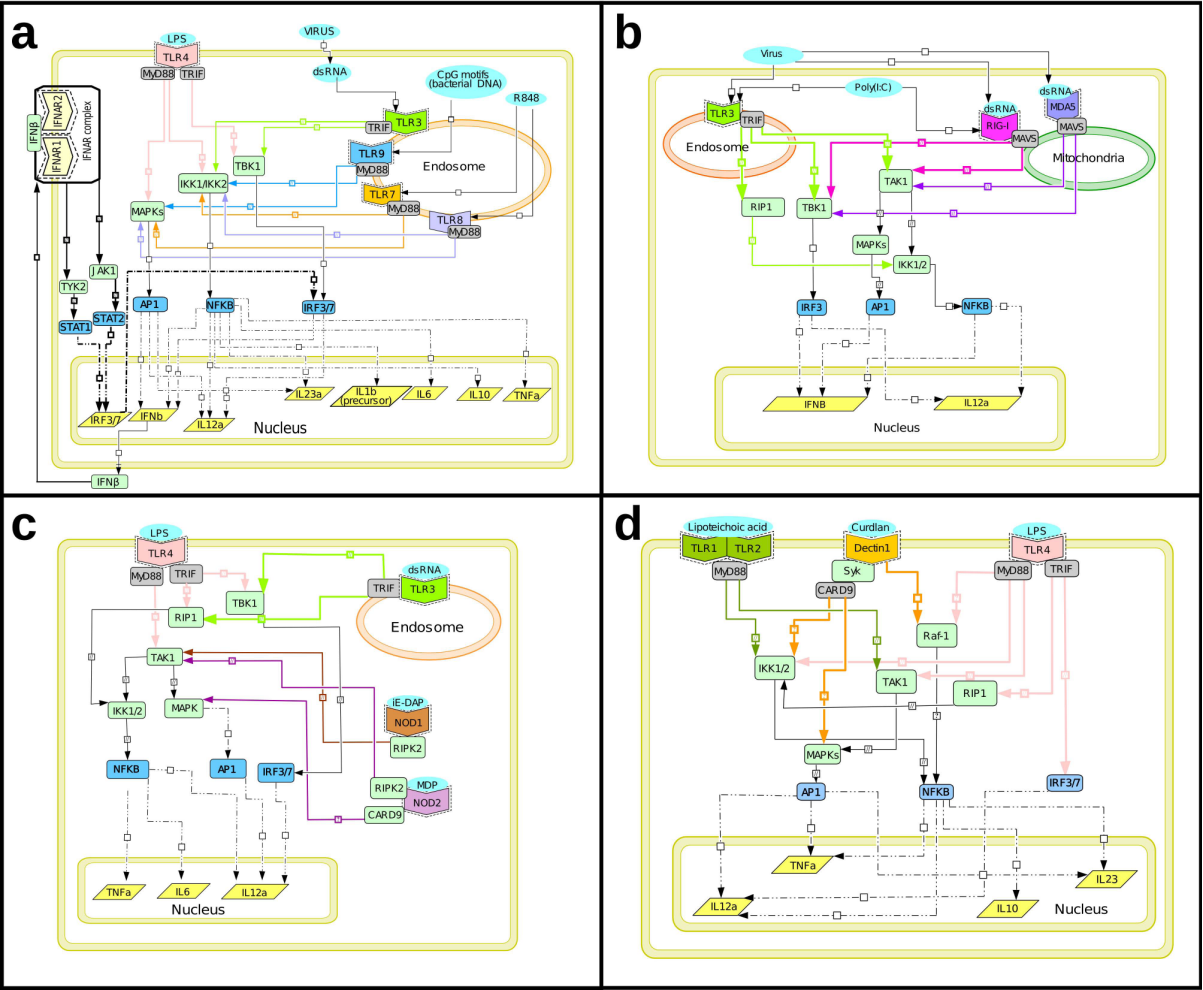
**Cross talks between signaling pathways downstream of PRRs**. **(a) **Cross talk between different TLRs. The diagram illustrates the cross talks between TLR3 and TLR 7-9, and between TLR4 and TLR7-9. For instance, TLR4 (in pink) and TLR7-9 (in yellow, light purple, and blue, respectively) signaling pathways converge at both IKKs and MAPKs. **(b) **Cross talk between TLR3 and RLRs (RIG-I and MDA5). **(c) **Cross talk between TLR4 and NLRs (NOD1 and NOD2). **(d) **Cross talks between TLRs and CLRs. The diagram illustrates the cross talks between TLR2 and Dectin-1, and between TLR4 and Dectin-1. Symbols: black or color-coded filled arrows, stimulatory reactions; black dashed and double-dotted lines, transcriptional activation reactions; black filled arrows with a bar, transport reactions. The presence of two forward slashes signifies that known stimulatory reactions (i.e. intermediate reactions) were omitted. PRR entities that are bordered with a dotted frame are considered in an active state.

**Figure 4:**
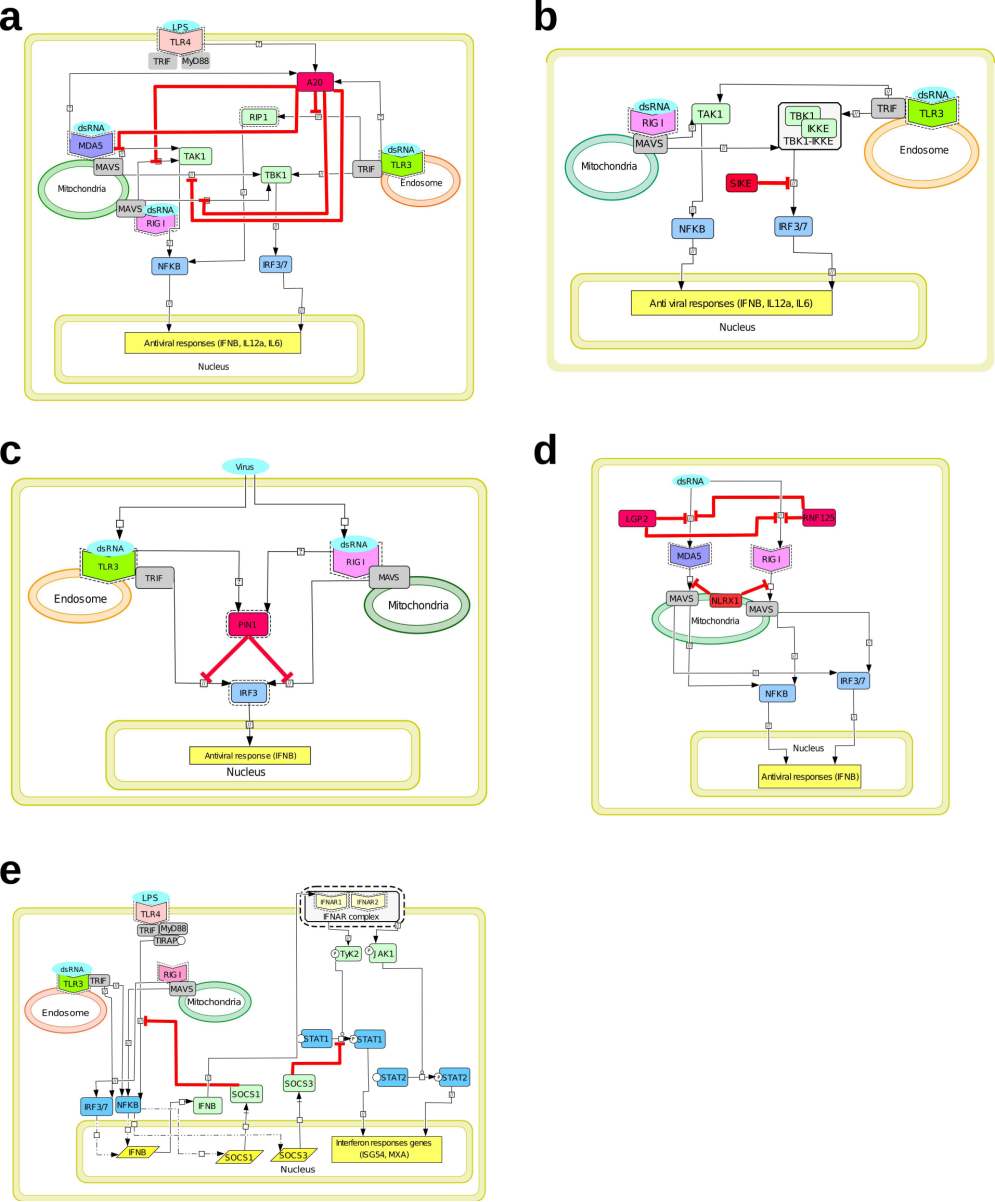
**Negative regulations of PRR responses**. **(a) **Negative regulation of TLR3-, RIG-I-, and MDA5-induced pathways by A20. **(b) **Negative regulation of RIG-I- and TLR3-induced pathways by SIKE. **(c) **Negative regulation of TLR3- and RIG-I-induced pathways by PIN1. **(d) **Negative regulation of MDA5 and RIG-I signaling by both RNF125 and NLRX1. **(e) **Negative regulation of JAK/STAT signaling by SOCS proteins, and of TLR4 signaling by SOCS1. In this diagram, transcriptional activation reactions were represented by black dashed and double-dotted lines, and protein phosphorylation was signified by P.

The DC signaling network (Figure 2) can be divided into four main pathways, each of which is activated by a different family of PRRs: the TLRs, CLRs, RLRs, and NLRs. Besides being initiated by different PRRs, these pathways involve distinct adaptors and lead to the expression of genes in response to individual microbes. Those genes encode inflammatory cytokines, such as interleukin-12 (IL-12, IL-6, IL-23), which are necessary to stimulate T cells (T helper cells Th1, Th2, Th17, or Tregs) for the adaptive immune response. Because T cell responses are beyond the scope of the DC signaling network, they were not illustrated on the DC map. Importantly, the pathways often share common downstream signaling molecules [17]. The pathway interconnectivity, which is illustrated on the DC map (Figure 2) and detailed further below, is presented in a more condensed form in Figure 3. Likewise, we describe diverse negative regulatory mechanisms employed by DCs to control their PRR responses to pathogens, and provide a basic depiction of those mechanisms in Figure 4.

There are 13 different members in the TLR family of proteins identified across mammalian species (TLR1-13). We omitted TLR10, which is not functional in mice, and TLR 12 and TLR13, which are lost in humans. TLRs recognize a wide variety of PAMPs ranging from lipids, lipoproteins, glucans to nucleic acids [18]. They are characterized by leucine rich repeats (LRRs) that mediate PAMP recognition and a cytoplasmic TIR domain that transmits the signal downstream via adaptor molecules. The main adaptors include MyD88, TRIF, TRAM and TIRAP [19-24]. Individual TLRs mediate distinct responses by association with a different combination of adaptor molecules. Additionally, some TLR family members can form heterodimers that recognize specific microbial structures. For instance, TLR1 and TLR6 were shown to form heterodimers with TLR2: TLR1/2 heterodimers interact with bacterial triacyl lipopeptides, while TLR2/6 heterodimers recognize diacyl lipopeptides and lipoteichoic acid [25,26]. While nearly all TLRs recruit MyD88, TLR3 mediates its response merely through TRIF. The association of TLRs and MyD88 recruits members of the IL-1 receptor-associated kinase (IRAK) family. In turn, IRAK4 and IRAK1 activate transcription factors NFκB and AP-1 through the canonical IKK complex and the MAPK pathway, respectively. NFκB consecutively stimulates the expression of inflammatory cytokine genes, including TNFα, IL-6 and IL-1β [3]. In contrast with MyD88, TRIF interacts with protein kinases IKKE and TBK1, which activate IRF3 and IRF7 [20,27]. Interestingly, TLR4 signals through both MyD88 and TRIF, leading to activation of NFκB and IRFs, respectively [20,28].

The RLRs RIG-I and MDA5 activate NFκB and IRF3 independently of TLRs. RIG-I interacts with either ssRNA or dsRNA through an RNA helicase domain, and recruits adaptor MAVS via a CARD-CARD association [29,30]. MAVS stimulates IKKE/TBK1, which in turn activate IRF3 and IRF7. Consecutively, those IRF family members induce type I IFN response by activating IFNβ transcription [20,31].

CLRs recognize carbohydrate structures present on bacterial or fungal pathogens (for review, see [32]). They are characterized by a carbohydrate recognition domain (CRD) involved in carbohydrate binding. Different CLRs, such as MR, Langerin, Dectin-1 and MGL, interact with different glucan structures. In our network diagram, we chose to depict the Dectin-1-dependent pathway in detail: Dectin-1 binds fungal β-glucans and recruits SYK and CARD9, which leads to activation of NFκB, and subsequent induction of inflammatory cytokines (for review, see [33]).

The NLR family of intracellular PRRs includes NOD1, NOD2, and NALP3. NOD1 and NOD2 recognize distinct motifs derived from the peptidoglycan bacterial cell wall, while NALP3 responds to multiple stimuli (for review, see [34]). NOD1 and NOD2 possess a CARD domain responsible for the signaling, whereas NALP3 has a pyrin domain instead (for review, see [35]). Both NOD1 and NOD2 recruit adaptor RICK, which activates TAK1. TAK1 activates the MAPK cascade and NFκB, which in turn induce genes of the IL-1 family of inflammatory cytokines. In response to diverse stimuli such as bacterial RNA or endogenous danger signals (extracellular ATP, uric acid crystals), NALP3 forms an inflammasome. The NALP3 inflammasome contains NALP3, ASC, Cardinal, and Caspase-1, and leads to activation of Caspase-1 and subsequent maturation of the pro-inflammatory cytokine IL-1β [36]. Here, we intentionally represented the NOD1- and NOD2-dependent pathways.

### Cross talk and synergy between the signaling pathways downstream of PRRs

Multiple PRRs can recognize a specific pathogen, thereby activating separate or shared pathways. Generally, the collaboration between PRRs typically results in enhanced transcriptional and cellular responses, i.e. synergistic inflammatory responses, as compared to each PRR. The fact that several receptors may be involved in pathogen recognition renders the system more robust against immune evasion by the pathogenic microbe. Not only can it scale up the inflammatory response, but it may also tailor it to the type of microbe encountered. Importantly, a better understanding of the effective interplay between DC signaling pathways may eventually be exploited in the development of vaccines. Hence, TLRs, RLRs, NLRs, and CLRs can cross talk and synergize to orchestrate immune responses effectively. Cross talks observed between PRRs that belong to the same family, or between different types of PRRs, are detailed below and on the DC map (Figure 2). Additionally, a reduced version of those cross talks is presented in Figure 3.

TLR3/4 synergize with endosomal TLR7-9 to increase the production of Th1- polarizing cytokine IL-12p70 by more than 20-fold compared to either receptor alone; the mechanisms underlying this synergy are not fully understood [37-39]. It is conceivable that activation of IRF transcription factors by dual TLR engagement may enhance IL-12 transcription. Additionally, TLR7 and TLR4 synergize for the production of inflammatory cytokine IL-1β [39]. Napolitani *et al*. speculated that synergistic TLR stimulation may result from sustained signaling, or possibly imply complementary signaling pathways that have yet to be identified [39]. The cross talk pathways that may contribute to TLR signaling synergy are illustrated in Figure 3a.

TLR3 and RLRs have been shown to be important in recognizing RNA viruses in different cellular compartments. TLR3 is present in endosomal membranes and binds to the dsRNA phagocytosed from viral infected apoptotic cells [40-42]. In contrast, RIG-I/MDA5 functions as a sensor that detects actively replicating viruses in the cytoplasm [43-46]. Although the pathways triggered by TLR3 and RLRs are independent, they converge further downstream and result in the activation of key transcription factors, such as NFκB, IRF3 and 7, and AP-1 (c-Jun/ATF2), which ultimately induce the expression of IFNβ [47,48] (Figure 3b). Recently, Perrot and his coworkers reported that concomitant engagement of TLR3 and RLR on myeloid DCs by poly(I:C) dsRNA is required to induce high levels of IL-12 and type I IFN, which in turn lead to an optimal production of IFNγ by NK cells [49].

Activation of NFκB and of the MAPK cascade occurs in the TLR and NLR signaling pathways (for review, see [34]) (Figure 3c). NOD2 agonist, MDP and TLR4 agonist, LPS have a synergistic effect on the production of inflammatory cytokine TNFα, which is apparently due to removal of a block in translation of the TNFα mRNA expressed in response to MDP [50]. NOD1 and NOD2 also act in synergy with TLRs 3, 4, and 9 in human DCs to induce IL-12p70 production and promote Th1 cell differentiation [51].

TLR2 and Dectin-1 are synergistic in mediating IL-12 and TNFα production [52] (Figure 3d). Interestingly, Dectin-1 can also promote synthesis of IL-2 and IL-10 in DCs through the recruitment of Syk kinase in response to zymosan, a cell-wall preparation of yeast [53]. Hence, during pathogenic infection, stimuli from either a single antigen or different antigenic components from a single pathogen could activate different PRRs [53]. The final immune response is the combined effect produced through activation of multiple PRRs and cross talks between the signaling pathways activated through these receptors.

### Network control and negative regulation of PRR responses

Appropriate and accurate PRR responses to pathogen signals are essential for the host defense. Yet over-activated cytokine responses to infection can be detrimental to the host. In order to generate an effective but non-toxic response, control mechanisms that negatively regulate the degree and duration of PRR responses are needed. Such surveillance mechanisms operate at different nodes in the PRR-induced signaling cascades. Regulatory mechanisms, which include degradation, sequestration, or inhibition of signaling molecules, are represented schematically in Figure 4. Proteins such as A20, SIKE and PIN1 negatively regulate both TLR and RLR signaling pathways, while others inhibit a specific pathway. For instance, LGP2 interferes with the recognition of viral RNA by RIG-I and MDA5. LGP2 is a RIG-I-like RNA helicase that sequesters dsRNA away from RIG-I. Thus, it acts as a negative regulator of RIG-I signaling. Unlike RIG-I and MDA5, LGP2 lacks the CARD domain required for the interaction with the MAVS adaptor and for signal transduction [54].

A20 functions as a feedback negative regulator of the TLR3 pathway by inhibiting TRIF-mediated induction of NFkB and IFNβ transcription (Figure 4a). It was demonstrated to interact with the adaptor protein TRIF by co-immunoprecipitation experiments, and targets it for degradation [55,56]. Moreover, A20 was shown to restrict TLR-induced TRAF6 ubiquitination, most likely by deconjugating ubiquitin chains onto TRAF6, thereby inhibiting the activation of NFκB signaling [57-60]. A20 was also shown to inhibit RIG-I-induced antiviral state, namely gene expression of IRF3, IRF7, and NFκB via its C-terminal ubiquitin ligase domain. According to Lin and collaborators, the inhibitory effect of A20 occurred upstream of the IKKE/TBK1 kinases, yet its biological target had yet to be identified [55]. It is noteworthy that A20 was previously shown to interact with TBK1 and IKKE by co-immunoprecipitation, thereby inhibiting IRF3 phosphorylation and subsequent dimerization; nevertheless, the molecular mechanism involved in this negative regulation by A20 was not entirely unraveled [61]. RIP1 is a key mediator of TLR3-induced NFκB activation [62]. Interestingly, A20 downregulates NFκB signaling through the cooperativity of its two ubiquitin-editing domains: one domain de-ubiquitinates RIP at Lys63, while the other poly-ubiquitinates RIP at Lys48, thereby targeting it for proteasomal degradation [60].

SIKE was demonstrated to associate with IKKE and TBK1 by co-immunoprecipitation (Figure 4b). Overexpression of SIKE inhibits IKKE- and TBK1-mediated antiviral response by disrupting their interaction with signaling components TRIF and IRF3. Indeed, those two kinases play a crucial role in RIG-I- and TLR3-triggered activation of IRF3 [63].

PIN1 is a peptidylprolyl cis-trans isomerase that promotes proteasomal degradation of phosphorylated IRF3 by augmenting its poly-ubiquitination [64] (Figure 4c). RNF125 is a ubiquitin ligase that negatively regulates RIG-I signaling, by targeting RIG-I for proteasomal degradation; likewise, RNF125 conjugates ubiquitin to MDA5 [65] (Figure 4d). NLRX1 is a potent inhibitor of the MAVS-mediated expression of the IFNβ gene (Figure 4d). It localizes to the mitochondrial outer membrane and disrupts the interaction between RIG-I and its adaptor MAVS [66].

SOCS proteins inhibit cytokine-mediated signal transduction by targeting JAK/STAT signaling (Figure 4e). Cytokines include various interleukins and IFNα/β/γ. Their transcription is induced by cytokine activity, which qualifies them as inducible feedback inhibitors. SOCS1 and SOCS3 inhibit JAK activity: SOCS1 binds to JAK2 and acts as a pseudo-substrate, while SOCS3 specifically binds to gp130 (signal transducer) cytokine receptors such as the IL-6 receptor (for review, see [67]). A study by Mansell and coll. indicated that SOCS1 may also poly-ubiquitinate and degrade TIRAP in TLR4 signaling, thereby resulting in an impaired NFκB response [68], whereas another report suggested that SOCS1 is part of a ubiquitin ligase complex, which directly interacts with NFκB in the nucleus, leading to its ubiquitination and degradation [69]. We chose to solely represent the interaction between SOCS1 and TIRAP. Concerning non-gp130 cytokine receptors like the IFNγ receptor, SOCS3 was shown to inhibit STAT1 phosphorylation [70].

Additional negative regulators include IRAK-M and phosphatases. IRAK-M negatively regulates TLR signaling by preventing the dissociation of IRAK1 and IRAK4 from MyD88 and the formation of IRAK1-TRAF6 complexes, as revealed by co-transfection experiments into heterologous cells followed by co-immunoprecipitation [71]. The authors proposed that IRAK-M may either inhibit IRAK1 and IRAK4 phosphorylation, or stabilize the TLR-MyD88-IRAK1-IRAK4 complex. Phosphatases were shown to be implicated in the regulation of PRR responses. Protein phosphatase 2C (PP2C) was demonstrated to dephosphorylate and thus inactivate TAK1 *in vitro*, and co-immunoprecipitation experiments indicated the association of PP2C with TAK1 [72,73]. Earlier studies demonstrated that PP2A plays a negative regulatory role in the activation of IKK and NFκB in response to various cellular stimuli [74,75]. However, more recent studies suggested a positive regulatory role, involving a physical interaction between PP2A and IKK [76,77].

### Computational motif detection analysis of the DC network

**Figure 5:**
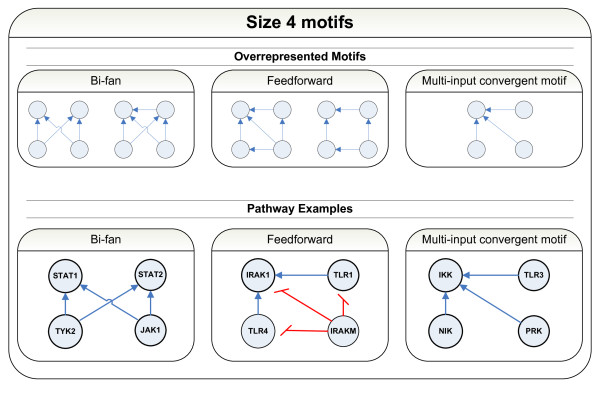
**Schematic representation of motifs and examples**. Upper panels: Overrepresented motifs; lower panels: network motif examples. Only motifs of 4 nodes are shown.

To identify motifs in our DC signaling network, we used a fully connected directed graph that was reconstructed from our DC map, as described in detail in Additional file 1, followed by a motif search using several detection methods [78-80]. We employed the FANMOD software [80] to identify motifs of size 3, 4, 5 and 6. Motifs with z-scores > 2 were considered as significantly overrepresented if obtained with a p-value < 0.005 in 1000 shuffled networks. No significantly overrepresented motifs of size 3 were detected. Among the significantly overrepresented motifs of size 4, 5 and 6, the bifan and the feedforward motifs were the top most enriched motifs detected (Additional file 2). The rest of the significantly overrepresented motifs contained generalizations of these two types as well as the multi-input convergence (MIC) motif. An example of each motif type is shown in Figure 5. Figure S2 in Additional file 1 illustrates instances of bifan motifs in the entire graph. Similar motifs of node size 5 and 6 were also found (data not shown). We observed that feedback motifs were not enriched in this network.

Motifs have been shown to functionally implement signal processing within larger networks [81,82]. Obtaining an outline of the regulatory motifs that are part of the DC network may provide further insight into this complex system. Bifan motifs, which support cross talk, and feedforward loops, which favor signal persistency and noise filtering have been shown to be prevalent in biological networks [78,83-85]. Overrepresentation of MIC motifs suggests another control mechanism whereby several routes lead to the regulation of the same target.

## Utility & Discussion

The DC pathway map is a public knowledgebase that offers a platform for the curation, diffusion, and update of information about the signaling cascades activated upon viral and bacterial infection in DCs. Its manual curation is based on extensive literature searches, and its dissemination relies on the availability of a navigable map of nodes and edges through the internet. Pathway layout has been optimized manually in order to provide more clarity to the diagram. In particular, we have put nodes that interact with one another in close proximity. In the web-published diagram, each node is clickable and links to a complete list of interactions in which it is involved; interactions themselves are supported by hyperlinked PMIDs. Moreover, each node that belongs to either a Protein or RNA category has one or more hyperlinked GeneIDs, and is tied to a wiki page, which lists all interactions in which it is engaged. Wiki pages allow experts to contribute their remarks, suggestions or literature updates. We expect to update the map regularly with new data derived from the literature as well as experts' intellectual contributions on the wiki. Hence, the DC map represents both an online resource and an opportunity for community-wide collaboration. An xml version of the pathway is available for download on the website to allow biologists to modify and expand it in accordance with their own experimental observations. This xml file (also available as Additional file 3) includes the PMIDs supporting interactions and GeneIDs describing entities.

Recently, Pico *et al*. created an open platform for the curation of biological pathways referred to as WikiPathways [86]. The use of wiki pages to represent the pathways allows limited access to the knowledgebase and does not support presentation of large networks, in contrast to our approach, which is to use the wiki to facilitate discussion.

Kitano's group previously created a comprehensive map of macrophage molecular interactions including ligands such as PAMPs and interleukins as input signals, and the release of cytokines and lipids as output signals [87]. In the present work, we included pathogens and PAMPs as inputs, as well as cytokines like IFNα and TNFα. As outputs, the induction of inflammatory cytokine expression, such as IL-6 and IL-12 was depicted. In their comprehensive map of TLR signaling, Kitano's group employed a top-down approach to build a model that emphasizes signal convergence [14]. We used a multi-centric approach to build the DC signaling network, beginning with the curation of induction of IFNβ enhanceosome through activation of the key transcription factors IRF3, NFκB, AP1 upon viral invasion. Parallel signaling shows cross talk at several points and network branches in many directions. In that respect, our approach is similar to that of Raza and coll. [88]. However, we also provide simplified diagrams that illustrate the major cross talk and negative regulatory mechanisms that are part of the DC signaling network. These schematics complement our comprehensive network by extracting the main information and presenting a simpler version of the complex mechanisms taking place in DCs.

This mechanism for generating specific pathways also improves upon the offerings that exist in such repositories as Science's STKE biological pathways database or KEGG. While STKE "pathway authorities" can be reached to with regards to pathway alterations, the process suffers from a lack of transparency and from the review article dilemma of being too biased in specificity or too general in the canonical pathways that may have stagnated in the repository. While repositories like Reactome and KEGG provide output in many usable formats for modelers, they lack transparency and focus despite the inclusion of specific reaction modules.

As we were finalizing this manuscript, a new community-based platform named Payao http://www.payaologue.org was developed by CellDesigner for sharing pathway models. Payao provides a web-based interface for adding tags and comments to curated pathway models. In contrast to our wiki system, it assigns privileges to specific community members [89]. As parallel approaches to the biocuration of pathway models may be taken, the research community is encouraged to share its knowledge and support curators in their efforts to assemble and edit pathway diagrams. The wiki system is robust and reliable in a sense that it keeps track of the changes made by contributors. As biocuration is gradually gaining more recognition, we reckon that our platform as well as others will each play a part in improving community-driven pathway enrichment [90].

With these tools to address the issues of curated network processes for use in modeling, we offer a curated signaling pathway of DCs undergoing viral infection. This pathway does not compare to current complexity and richness of Kitano's Toll-like receptor network [14]. However, manual curation from our community has produced an easily parsable network with the possibility of scaling for unforeseen future applications. The high degree of curation in this network offers a framework for incorporation of private or public experimental data as well as well represented evidence to enhance the validity in modeling. As a community-driven process, we hope that specific networks can rapidly grow and overcome the hurdles of increased data saturation and complexity, while meeting the needs of experimentalists, modelers and computational biologists.

We performed an automated motif detection analysis of the entire DC network. This analysis revealed an over-representation of motifs favoring mechanisms such as cross talk, signaling persistence, and signal convergence. In particular, the families of bifan, feedforward and MIC motifs were enriched. Bifans allow for cross talk between the different regulators and may be an important control mechanism for activating the same pathway by multiple types of triggers. The feedforward loops allow for noise reduction from transient activation of the general regulator and are effective only once there are enough signals to activate the specific regulator [78,83-85]. Finally, the MIC motif also provides another variation of the bifan control mechanism whereby several pathways lead to the regulation of the same target. As DCs need to coordinate signals triggered by various pathogens to produce the specific immune response, the prevalence of bifan, feedforward and MIC motifs may be providing DCs with mechanisms to support their proper function.

## Conclusions

In this report, we present a comprehensive network map of the DC signaling network. Based on a detailed and thorough search of the relevant literature, we manually curated a pathway map of the signaling events triggered upon viral and bacterial infection. In addition to TLR-dependent pathways, cascades deriving from CLRs, RLRs and NLRs are depicted. The map is intended to be comprehensive and help researchers to unravel the signal transduction pathway and gene response mechanisms occurring in the DC in response to pathogens. We anticipate updating the map regularly using data drawn from newly published studies, as well as through exchanges with researchers whose area of expertise is cellular and molecular immunology. Those exchanges will be made possible by the availability of a wiki, which will let experts suggest corrections or additions through their feedback and comments.

## Availability and requirements

The DC signaling pathway map is accessible at http://tsb.mssm.edu/pathwayPublisher/DC_pathway/DC_pathway_index.html.

## Abbreviations

AP-1: activator protein-1; APC: antigen-presenting cell; ATF2: activation transcription factor 2; BioPP: Biological Pathway Publisher; CARD: caspase recruitment domain; CLR: C-Type lectin receptor; CRD: carbohydrate recognition domain; DC, Dendritic cell; dsRNA: double-stranded RNA; IFN: interferon; IKK: IκB (inhibitor of κB) kinase; IL: interleukin; IRAK: IL-1 receptor-associated kinase; IRF: IFN regulatory factor; IκB: inhibitor of κB; KEGG: Kyoto Encyclopedia of Genes and Genomes; LPS: lipopolysaccharide; LRR: leucine rich repeat; MAPK: mitogen-activated protein kinase; MAVS: mitochondrial associated viral stimulator; MDA5: melanoma differentiation associated gene 5; MDP: muramyl dipeptide; MGL: macrophage galactose-type lectin; MHC: major histocompatibility complex; MIC: multi-input convergence; MR: mannose receptor; MyD88: myeloid differentiation primary response gene 88; NALP3: NLR family, pyrin domain containing 3; NFκB: nuclear factor kappa B; NLR: nucleotide-binding oligomerization domain (NOD)-like receptor; NLRX1: NLR family member X1; NOD: nucleotide-binding oligomerization domain; PAMP: pathogen-associated molecular pattern; PIN1: peptidylprolyl cis/trans isomerase, NIMA (never in mitosis gene a)-interacting 1; PMID: PubMed ID; PRR: pattern recognition receptor; RICK: RIP (receptor-interacting protein)-like interacting caspase-like apoptosis regulatory protein kinase; RIG-I: retinoic acid-inducible gene-I; RIP1: receptor interacting protein 1; RLR: RIG-I-like receptor; RNF125: Ring finger protein 125; SBML: Systems Biology Mark-up Language; SIKE: Suppressor of IKKE; SOCS: suppressor of cytokine signaling; ssRNA: single-stranded RNA; STKE: Signal Transduction Knowledge Environment; SYK: spleen tyrosine kinase; TAK1: transforming growth factor β-activated kinase 1; TBK1: TANK (TNFR-associated factor family member-associated NFkB activator) binding kinase 1; TIR: Toll/IL-1R; TIRAP: TIR domain-containing adaptor protein; TLR: Toll-like receptor; TNFα: tumor necrosis factor-α; TRAM: TRIF-related adaptor molecule; TRIF: TIR-containing adaptor inducing IFNβ.

## Authors' contributions

SP was the initial curator and wrote the first manuscript draft. HP is the present curator and revised the manuscript. JS contributed to the curation, figure design and manuscript preparation. GN maintained and revised the custom software suite utilized for web-publishing. GN conceived and designed the network motif analysis. IN and GN performed the network motif analysis and participated in the manuscript writing. SCS organized and directed the project and revised the manuscript. All authors read and approved the final version.

## Supplementary Material

Additional file 1 **Supporting Methods & Figures S1-S2**. This file describes the method employed for the computational motif detection analysis of the DC network. It also illustrates examples of motifs identified in the DC network in Figure S1, and instances of bifan motifs found in the entire graph in Figure S2. Click here for file

Additional file 2 **FANMOD size 4 output**. This output file, obtained using the FANMOD program, contains all overrepresented motifs of size 4. Click here for file

Additional file 3 **DC signaling pathway**. This file is an xml version of the DC signaling pathway map, which allows biologists to edit and/or expand it in accordance with their own experimental observations. Click here for file
