# Supplementary material for: Signaling network of dendritic cells in response to pathogens: a community-input supported knowledgebase
Source: BMC Syst Biol. 2010 Oct 7;4:137. doi: 10.1186/1752-0509-4-137 (PMC2958907; doi:10.1186/1752-0509-4-137)
Supplement: Additional file 1 — Supporting Methods & Figures S1-S2. This file describes the method employed for the computational motif detection analysis of the DC network. It also illustrates examples of motifs identified in the DC network in Figure S1, and instances of bifan motifs found in the entire graph in Figure S2. [file 1752-0509-4-137-S1.DOC]

**Supplementary Online Materials for:**

Signaling Network of Dendritic Cells in Response to Pathogens: A Community-Input Supported Knowledgebase

**Contents**

Methods

Supporting Figures S1- S2

Supplementary File list

References

**Methods**

Constructing the network:

We used our curated DC map to construct a fully connected directed graph. We applied consistent rules to represent pathway reactions into network made of 202 nodes and 305 edges. The edges were drawn from reactants to products, except in the case of modifiers. For the reactions that had modifiers, the edges were drawn from the modifier to the reactant and from the modifier to the product. The reasoning for this was that the reaction proceeds by the modifier exerting an effect both on the reactant and the product.

The python libraries libsbml [1], networkx [2] and graphviz [3] were used for network construction and visualization.

**Fig. S1**. Schematic representation of the motifs of size 5 and 6 identified within the network.

Network motifs identified using the FANMOD [4] program. The figure visually represents the text based output, produced by the FANMOD program. These motifs are among the most statistically significant identified network motifs of size 4 within the network (compared to 1000 shuffled networks).

**Fig. S2**. Visualization of the Bifan motif (using the frequency concept f1) overplayed on the entire graph.

Network motifs identified using the MAVisto [5] program. These motifs are the most statistically significant identified network motifs of size 4 within the network (compared to 1000 shuffled networks). The MAVisto program searches for motifs in directed networks, it does not distinguish between positive and negative links. Thus, for this analysis we considered positive and negative links as unidirectional.

**Supplementary File list**

DC signaling pathway map in xml format - SupplData_DCsignaling_13aug.xml

FANMOD size 4 output - DCgraph.txt.OUT

**References**

1. Bornstein BJ, Keating SM, Jouraku A, Hucka M: **LibSBML: an API library for SBML**. *Bioinformatics* 2008, **24**(6):880-881.

2. Hagberg AA, Schult, DA, Swart PJ: **Exploring Network Structure, Dynamics, and Function using NetworkX**. In: *Proceedings of the 7th Python in Science conference (SciPy 2008): 2008; Pasadena, CA USA*; 2008: 11-15

3. North ERGaSC: **An open graph visualization system and its applications**

**to software engineering**. *SOFTWARE—PRACTICE AND EXPERIENCE* 2000, **30**(00(S1)):1203--1233.

4. Wernicke S, Rasche F: **FANMOD: a tool for fast network motif detection**. *Bioinformatics* 2006, **22**(9):1152-1153.

5. Schreiber F, Schwobbermeyer H: **MAVisto: a tool for the exploration of network motifs**. *Bioinformatics* 2005, **21**(17):3572-3574.
